# Sea ice breakup and marine melt of a retreating tidewater outlet glacier in northeast Greenland (81°N)

**DOI:** 10.1038/s41598-017-05089-3

**Published:** 2017-07-10

**Authors:** Jørgen Bendtsen, John Mortensen, Kunuk Lennert, Jens K. Ehn, Wieter Boone, Virginie Galindo, Yu-bin Hu, Igor A. Dmitrenko, Sergei A. Kirillov, Kristian K. Kjeldsen, Yngve Kristoffersen, David G. Barber, Søren Rysgaard

**Affiliations:** 1 Arctic Research Centre, Aarhus University, 8000 Aarhus, Denmark; 2 ClimateLab, Symbion Science Park, Fruebjergvej 3, 2100 Copenhagen O, Denmark; 3 Greenland Climate Research Centre, Greenland Institute of Natural Resources, PO Box 570, 3900 Nuuk, Greenland; 4 Centre for Earth Observation Science, CHR Faculty of Environment, Earth, and Resources, University of Manitoba, 499 Wallace Building, Winnipeg, MB R3T 2N2 Canada; 5 Department of Earth Sciences, University of Ottawa, Ottawa, Ontario K1N 6N5 Canada; 6 Centre for GeoGenetics, Natural History Museum, University of Copenhagen, Øster Voldgade 5-7, 1350 Copenhagen K, Denmark; 7 Department of Earth Science, University of Bergen, Bergen, Norway

## Abstract

Rising temperatures in the Arctic cause accelerated mass loss from the Greenland Ice Sheet and reduced sea ice cover. Tidewater outlet glaciers represent direct connections between glaciers and the ocean where melt rates at the ice-ocean interface are influenced by ocean temperature and circulation. However, few measurements exist near outlet glaciers from the northern coast towards the Arctic Ocean that has remained nearly permanently ice covered. Here we present hydrographic measurements along the terminus of a major retreating tidewater outlet glacier from Flade Isblink Ice Cap. We show that the region is characterized by a relatively large change of the seasonal freshwater content, corresponding to ~2 m of freshwater, and that solar heating during the short open water period results in surface layer temperatures above 1 °C. Observations of temperature and salinity supported that the outlet glacier is a floating ice shelf with near-glacial subsurface temperatures at the freezing point. Melting from the surface layer significantly influenced the ice foot morphology of the glacier terminus. Hence, melting of the tidewater outlet glacier was found to be critically dependent on the retreat of sea ice adjacent to the terminus and the duration of open water.

## Introduction

The Greenland Ice Sheet mass loss has accelerated^1, 2^ and a better understanding of processes involved in this speed-up of glacial melt and ice discharge is therefore required. Of particular interest is the more than 200 tidewater outlet glaciers around Greenland^3^ because their direct contact with coastal water masses constitute a link between ocean heat transport, sea ice and melt of glacial ice^4^. However, coastal water masses around Greenland vary considerably between the east and west coast^5^ and also along the climatic gradient from southern to northern Greenland (i.e. a latitude interval of more than 23 degrees).

Relatively warm water masses have been shown to be in contact with tidewater outlet glaciers along the eastern^6, 7^ and western^8, 9^ coasts of Greenland where they provide energy for subsurface melt^10, 11^. Tidewater outlet glaciers at high polar latitudes have been studied at the floating ice tongue of Nioghalvfjerdsfjorden (79°N) and melting of glacier ice has been explained by heat from deeper and warmer (>0 °C, depth >150 m) recirculated Atlantic water^12^. Studies of the floating ice shelf in the Peterman Fjord (81°N, located in Nares Strait) also showed that subsurface melting could account for basal melt of the ice shelf^13^. Other studies of polar tidewater outlet glaciers include studies at Svalbard (~79°N) where the influence from ocean heat transport was manifested by a strong correlation between calving activity and subsurface ocean temperatures^14^. These studies elucidate the important role of sub-surface ocean heat transport for melting glacial ice.

However, other processes also affect terminus stability. For instance, an analysis of satellite images showed that changes in atmospheric conditions only had a minor influence on terminus position of five large outlet glaciers and, therefore, it was hypothesized that other processes such as ice thickness, subglacial topography and fjord bathymetry may regulate the retreat or advance of these glaciers^15^. Ice mélange and a protecting fast ice cover in front of glacier termini have been shown to influence the stability of the floating ice shelf in Nioghalvfjerdsfjorden on decadal to millenial time scales^16^ and recent speed-up and mass loss of both Nioghalvfjerdsfjorden and Zachariae Isstrøm occurred in periods with increased air temperature and low concentrations of sea ice and further enhanced by entry of warm subsurface-temperatures^17^. Retreats of the major outlet glacier at Jakobshavn Isbræ have also been related to a weakening of the ice mélange in front of the terminus^18, 19^. The Ellesmere Island ice shelves, facing the Arctic Ocean, have decreased significantly during the last century^20^ and, recently, a large fraction of the remaining shelves calved during summer 2008^21^. Calving events of the Ellesmere Island ice shelves have also been associated with longer periods of open water and loose pack ice along shelf margins^22^. Such conditions increase surface melt, wave erosion and removal of a resisting pressure on calving ice from surrounding land-fast ice and, thereby, promote fast disintegration of the glacier tongue^23^. Thus, these observations indicate the important role of sea ice cover and ocean heat transport in front of the glaciers.

**Figure 1:**
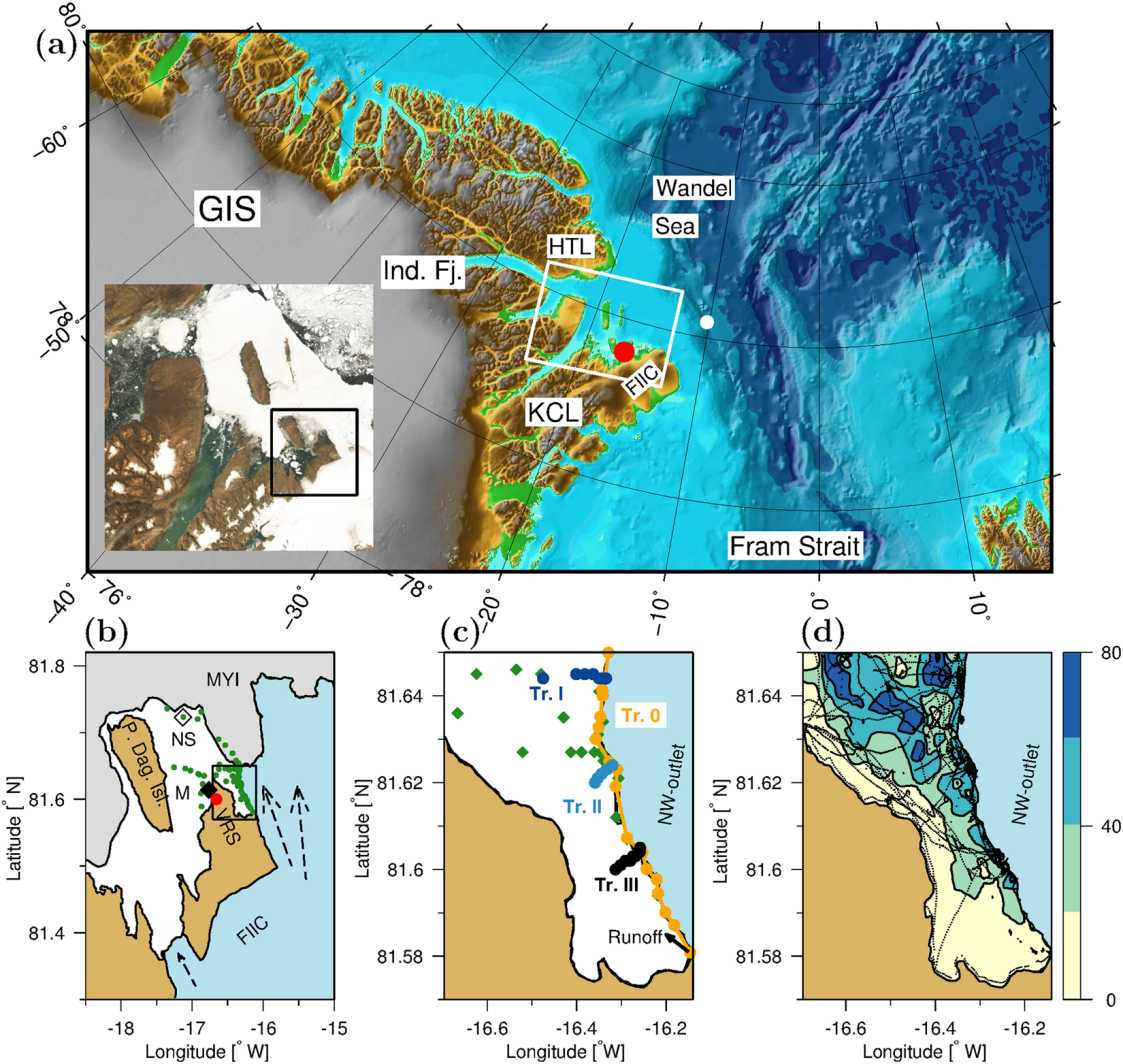
Study area and station map. (**a**) Northeastern Greenland and the Greenland Ice Sheet (GIS), Independence Fjord (Ind. Fj.) and Herluf Trolle Land (HTL), north of the fjord system, the peninsula Kronprins Christian Land where the Villum Research Station (VRS) is located (red bullet) next to Flade Isblink ice cap (FIIC). Station YK175 is marked on the shelf (white bullet). The map is based on data from IBCAO^43^ and produced by the GMT^45^ software. Landsat 8 satellite image from 12 August 2015 is shown in the inset (white rectangle). Landsat 8 data was compiled by the U.S. Geological Survey; Data processed by GDAL - Geospatial Data Abstraction Library: Version 1.10.1, Open Source Geospatial Foundation, http://gdal.osgeo.org). (**b**) Digitized satellite image (black rectangle shown on inset in (**a**)) and CTD-stations (green symbols). The CTD-station NS (open diamond, 81°43.376′N, 17°7.736′W) located northwest of the terminus at the multi-year ice edge (MYI) and the CTD-mooring located at station M (black diamond) between Prinsesse Dagmar Island and VRS. Dashed arrows indicate glacier ice flow (QGIS was applied for digitizing, Quantum GIS Development Team (2015). Quantum GIS Geographic Information System. Open Source Geospatial Foundation Project. http://qgis.osgeo.org. The maps (**b**–**d**) were made by the scientific graphic language Gri, http://gri.sourceforge.net). (**c**) Station map along the terminus (black rectangle in panel b) with stations shown along transect 0 (orange), I (blue), II (light blue), III (black) and the remaining stations in the area (green diamonds) between the north-western glacier outlet (NW-outlet) and VRS. Runoff from a meltwater river along the glacier on land is indicated (black arrow). (**d**) Bathymetry (m) shown by contours and colors obtained from echo soundings (dotted lines).

The ocean circulation offshore northeast Greenland between ~79°N–83°N is characterized by a southward current parallel to the shelf, in accordance with the general ice drift in the area^24^. Thus, coastal and shelf water masses in the western Fram Strait are generally transported southward from the Wandel Sea (Fig. 1), and are mainly comprised of Polar Surface water in the upper ~100 m with warmer (>0 °C) water of Atlantic origin below 200 m^25^. However, few oceanographic observations have been obtained near tidewater outlet glaciers from the north coast of Greenland linking ocean circulation and the ice-ocean interface, and critical processes for melting of these glaciers towards the Arctic Ocean are, therefore, currently unknown.

In this study, we present hydrographic measurements from the northeastern coast of Greenland at a tidewater outlet glacier from the largest peripheral ice cap in Greenland^26, 27^, Flade Isblink Ice Cap (FIIC). In addition, we analyze water masses near the terminus and argue that the terminus is a floating ice shelf. The observed ice foot morphology at the terminus is related to large melt rates in the surface layer, and we show that the short open water period has a major influence on the heat budget near the outlet. Finally, our results are used to explain the observed decadal variation of the outlet glacier.

## Study area at Flade Isblink Ice Cap

The fjord complex in northeastern Greenland associated with Independence fjord (Ind. Fj., Fig. 1a), Hagen fjord and Denmark fjord, connects shelf and coastal water masses with the Greenland Ice Sheet (GIS) through marine and land-terminating outlet glaciers. Outflow from the fjords occurs between Herluf Trolle Land (HTL) and the peninsula located at the northern Kronprins Christian Land (KCL).

The newly established Villum Research Station (VRS, red bullet in Fig. 1) provided logistic support for the investigations in this remote area, which is inaccessible from the sea side due to multiyear sea ice (MYI). Our study in August 2015 was the first open water investigation of the hydrographic conditions around FIIC. Surface conditions during late summer were characterized by a relatively thin (<1 m) and dynamic sea ice floe field around VRS and a thicker MYI coverage further offshore. Sea ice around the station broke up during the first week of August 2015 and thereafter a part of the terminus of FIIC was accessible by boats. Satellite imagery (Landsat 8) from 12 August 2015 showed that MYI covered the area from FIIC and across the mouth of the fjord system to HTL (Fig. 1a, inset). Comparison between the MYI edge, digitized on the satellite imagery (30 m resolution), and measurements along the MYI edge carried out on 21 August showed that the MYI edge had moved ~1 km northward during 9 days (Fig. 1b, bullets on MYI).

The bathymetric measurements conducted west of the northwestern outlet glacier (referred to as NW-outlet, Fig. 1c,d) showed a relatively shallow area with depths less than 20 m near land and a gradual deepening (to ~60 m) towards the terminus (Fig. 1d). The steep slope observed near the terminus was likely due to previous wider extensions of the ice cap in the bay (e.g. Fig. S1e).

## Results

### Measurements at Flade Isblink Ice Cap

**Figure 2:**
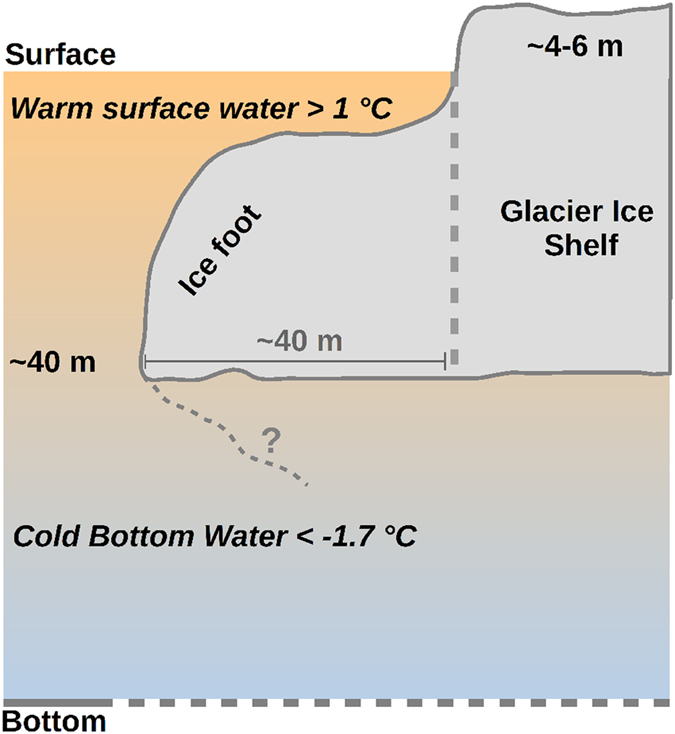
Ice foot at the terminus. Conceptual illustration of the terminus at the NW-outlet ice shelf from Flade Isblink ice cap showing an ice foot of about 40 m deep and a height of 4–6 m above sea level (observed ice foot shapes are shown in Fig. 5e). The vertical dashed line show the terminus after calving of the ice foot. The question mark below the ice foot indicate the unknown shape (dashed line) below the glacier.

Measurements were made at the NW-outlet from 10 to 25 August 2015. Relatively calm conditions, interrupted by a few days with strong winds, prevailed during the study period. No indications of subglacial discharge were observed while a noticed low calving activity made it possible to perform measurements close to the glacier. Several measurements were obtained within 1 m from the glacier terminus. An ice foot was present at some locations along the terminus (Fig. 2) whereas at other locations the bottom depth ranged between 50–70 m at the ice front.

**Figure 3:**
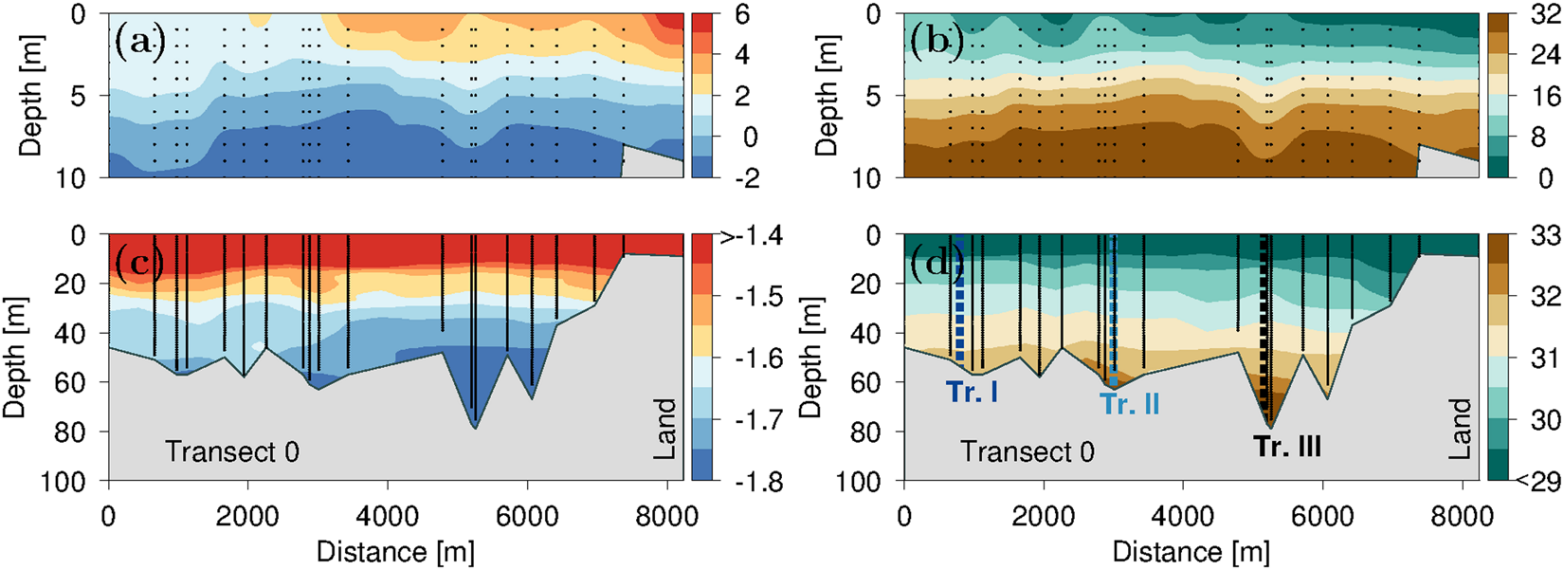
Transect 0 along the terminus. (**a**) Temperature in the upper 10 m, (**c**) in the whole water column and (**b**) salinity in the upper 10 m and (**d**) in the whole water column. Note the varying color scales. The location of Transect I-III are indicated with dashed lines in (**d**). Stations were collected in the period 10–25 August and are indicated with vertical dotted lines.

Near-glacier CTD-profiles from the entire period were sampled on a transect along the glacier front (transect 0, Fig. 1c). Only profiles that reached deeper than 20 m were considered on this transect 0. Three transects were sampled perpendicular to the glacier front (Figs 3 and S1).

Transect I was furthest from land and ~800 m from the MYI edge. The first profile was achieved 2 m from the terminus where the depth to the ice foot was 6 m and the height of the glacier was 4–6 m above sea level. The depth of the ice foot increased gradually to 36 m at 15 m from the terminus, where the depth increased abruptly to 53 m according to echo sounder readings. CTD-profiles achieved within the first ~15 m from the terminus of the glacier reached the ice bottom and were then sliding along the steeply sloping surface of the ice foot. Transect II was achieved ~5 km from land and the nearest profile was ~70 m from the terminus. Echo soundings near the glacier (within a distance of ~10 m) showed that the glacier front continued vertically towards the bottom with no signs of an ice foot. Together with visual observations of ‘fresh’ glacier ice above sea level these observations indicated that calving had occurred recently at this location. We witnessed several occasions of calving. The bottom depth was about 60 m along this transect (Fig. S1c,d). Transect III was located about 5 km from the MYI and ~3 km from land. The ice foot surface at the glacier front was located at about 2 m depth and it increased to 35 m at a distance of 38 m from the glacier, after which the depth increased abruptly to 73 m.

### Water masses at the terminus

Water masses near the terminus were analyzed by combining all deep stations collected close to the terminus in the period 10–25 August (Fig. 3). Surface temperature increased towards land from 1.3 °C at the MYI edge to 2.0 °C near the coast while salinity decreased from 9.1 to 4.2 (practical salinity units, Methods). The low salinity at the innermost station could be explained by a nearby turbid meltwater river flowing along the glacier on land (Fig. 1c). The upper water column was characterized by a shallow thermo- and halocline between 5 and 10 m.

The bottom water temperatures at a depth of 44 m along the terminus decreased from −1.68 °C at the MYI edge to the coldest temperatures at transect III of −1.74 °C (Fig. 3a), i.e. a significant temperature decrease towards land. In addition, the coldest bottom water temperature (−1.81 °C) and a high salinity (32.93) were observed in the deepest area of transect III at 75 m (Fig. S1e).

A very low temperature of −1.807 °C (S = 32.439, P = 60 dbar), i.e. 0.014 °C above *in situ* freezing temperature, was observed during a deep profile (bottom depth 70 m) taken ~50 m from the glacier terminus on transect III. Low temperatures were also observed between 45–57 m of the water column, where *in situ* temperatures were within 0.005 °C of the freezing point and where super-cooling of up to 0.002 °C was observed in the layer between 48 and 50 m. Note that the super-cooling was within the instrument uncertainty range of ±0.005 °C. However, such low temperatures strongly indicated that subsurface freezing had occurred at the glacier.

**Figure 4:**
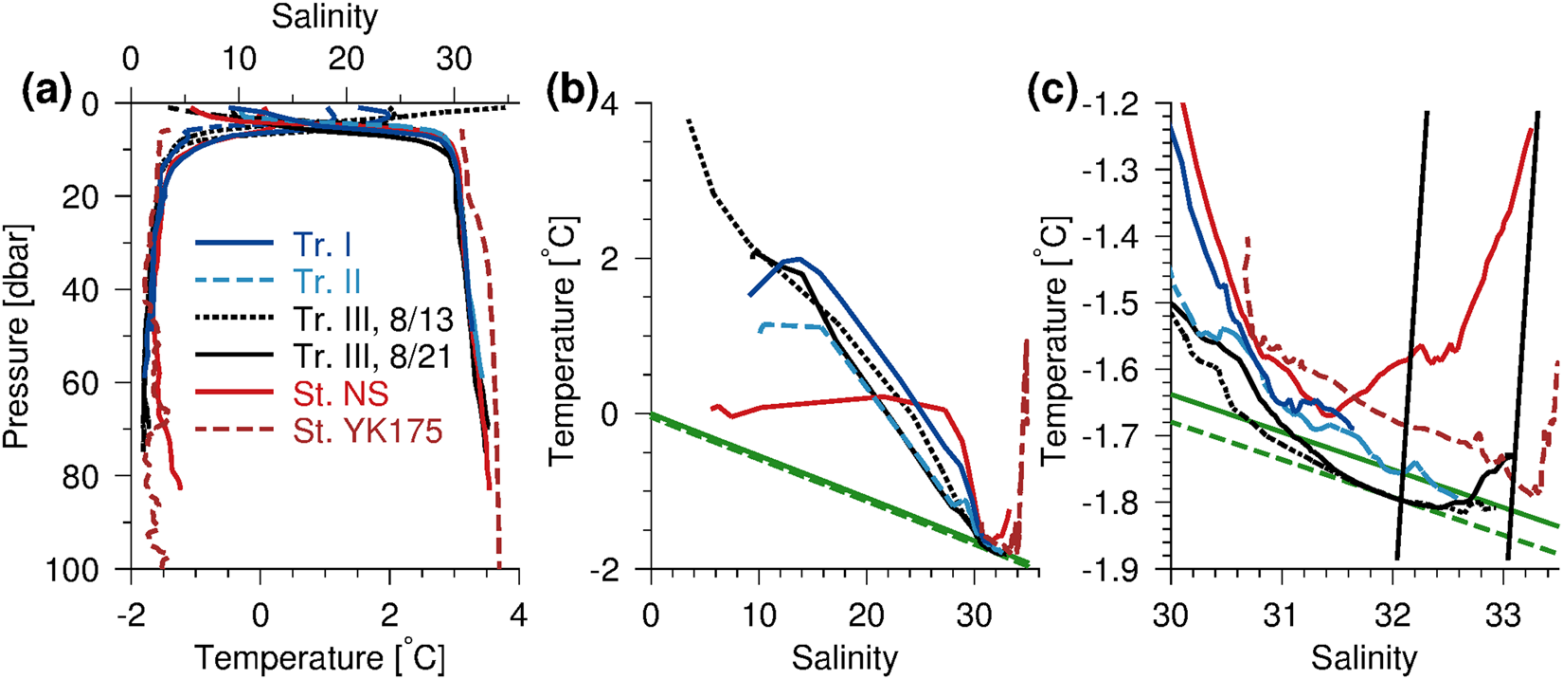
TS-diagrams. (**a**) Temperature and salinity profiles near terminus at transect I (blue), transect II (dashed lightblue), transect III (black dashed (13 Aug.) and solid (21 Aug.) lines), station NS (red line) and on the shelf (YK175, brown dashed line). (**b**) TS-diagram and (**c**) for S > 30. Meltlines (black near-vertical lines) and lines of freezing temperature are shown for P = 0 dbar (green) and P = 55 dbar (dashed green), respectively.

Water properties at the bottom of the deep profile at transect III (T = −1.73 °C, S = 33.02, P = 70 dbar) showed that the highest salinity along the terminus was found at this deepest location and temperature was well above the *in situ* freezing temperature of −1.86 °C (Fig. 4c). High salinities were also found at the bottom of transect II (T = −1.78 °C, S = 32.41, P = 57 dbar) with temperatures near the freezing point between 39 and 57 m. Bottom water properties were slightly warmer and less saline on transect I (T = −1.71 °C, S = 31.73, P = 50 dbar) with lower bottom salinities further from the glacier (Fig. S1). These observations show water masses with temperatures at or near the freezing point close to the terminus.

### Tracing bottom water masses at the terminus

The origin of bottom water at the terminus was traced by comparing bottom temperature and salinity with surrounding stations. The station with the highest bottom temperature and salinity (T = −1.24 °C, S = 33.25, P = 83 dbar) was located at station NS northwest of the terminus at the MYI edge and both T and S were significantly higher than observed at the terminus (Fig. 4). This could be explained as cooling and freshening of this water mass due to melting of ice. Melt lines (near-vertical lines shown in Fig. 4c), defined from the bottom water properties^28^ and with only latent heat exchange and freshwater dilution considered, indicated a cooling of ~0.3–0.5 °C of the bottom water mass at transect II and III. The near-glacial profile at transect III showed a layer between 35 and 60 m with *in situ* freezing temperatures located above a warmer and more saline bottom water mass (Fig. 4c). A visit to the same station one week earlier revealed significantly lower bottom water temperatures (Fig. 4c, black dashed line). These observations showed that short-term variability modified the bottom water at the terminus where cold water had been replaced by warmer water during the week.

### Annual variation of shallow subsurface water near FIIC

**Figure 5:**
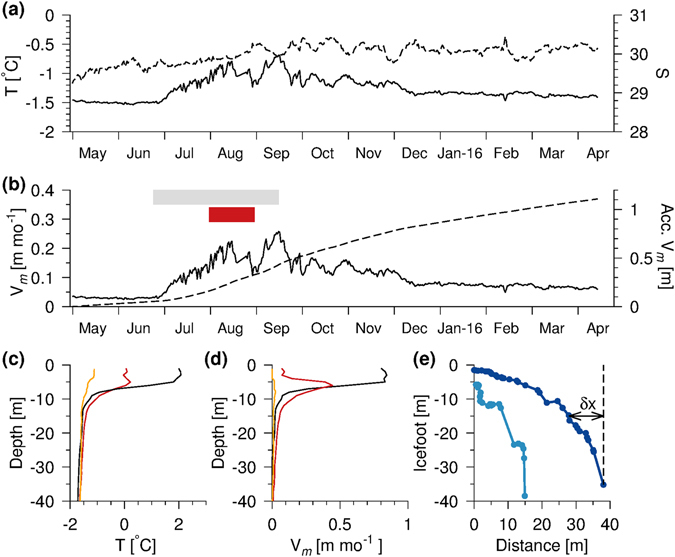
Melt rates and icefoot. (**a**) Temperature (solid line) and salinity (dashed, triangle) from mooring at station M. (**b**) Melt rate and accumulated melt during the periods where duration of ice breakup (gray bar) and open water in front of the terminus (red bar) are indicated. (**c**) Temperature (transect III, black), station NS (red) and a profile from April 2015 (orange), (**d**) the corresponding melt rates and (**e**) observed ice foot (transect I, light blue; transect III, blue) and glacier melt (δx).

Continuous measurements from the CTD-mooring deployed at 13.5 m, St. M, (Fig. 1b) from May 2015 to April 2016 showed the seasonal variations of temperature and salinity in the shallow sub-surface water masses surrounding the terminus at FIIC. Monthly averaged temperature and salinity increased from −1.49 °C to −0.97 °C and 29.54 to 29.95 between May and August 2015 (Fig. 5a). During the fall, the water temperature decreased gradually from a maximum value of −0.69 °C in September to a monthly average of −1.31 °C (S = 30.11) in December. Thereafter it remained relatively constant until April 2016.

## Discussion

### Dynamics of the FIIC NW-outlet

FIIC covers an area of 8,500 km^2^ and is likely formed after the Holocene climatic optimum^29^. The main north flowing outlet of the ice cap is a ~25 km broad outlet located east of VRS (Fig. 1b). This part of the ice cap has previously been described to have a floating tongue of up to 20 km long and only a small change of the glacier terminus position occurred from 1961 to 1978^30^. Surface slope of the glacier, derived from satellite data, showed that the easternmost part of the glacier, i.e. outside of our study area, was a floating ice shelf^31^. However, whether the portion located nearest VRS, i.e. the NW-outlet glacier investigated here, was floating, could not be concluded from this study. We analyzed a 1978 surface elevation profile of the outlet glacier and this revealed a relatively flat surface for more than 20 km along the terminus, and this would be expected if the outlet had a floating tongue (Fig. S2a).

**Figure 6:**
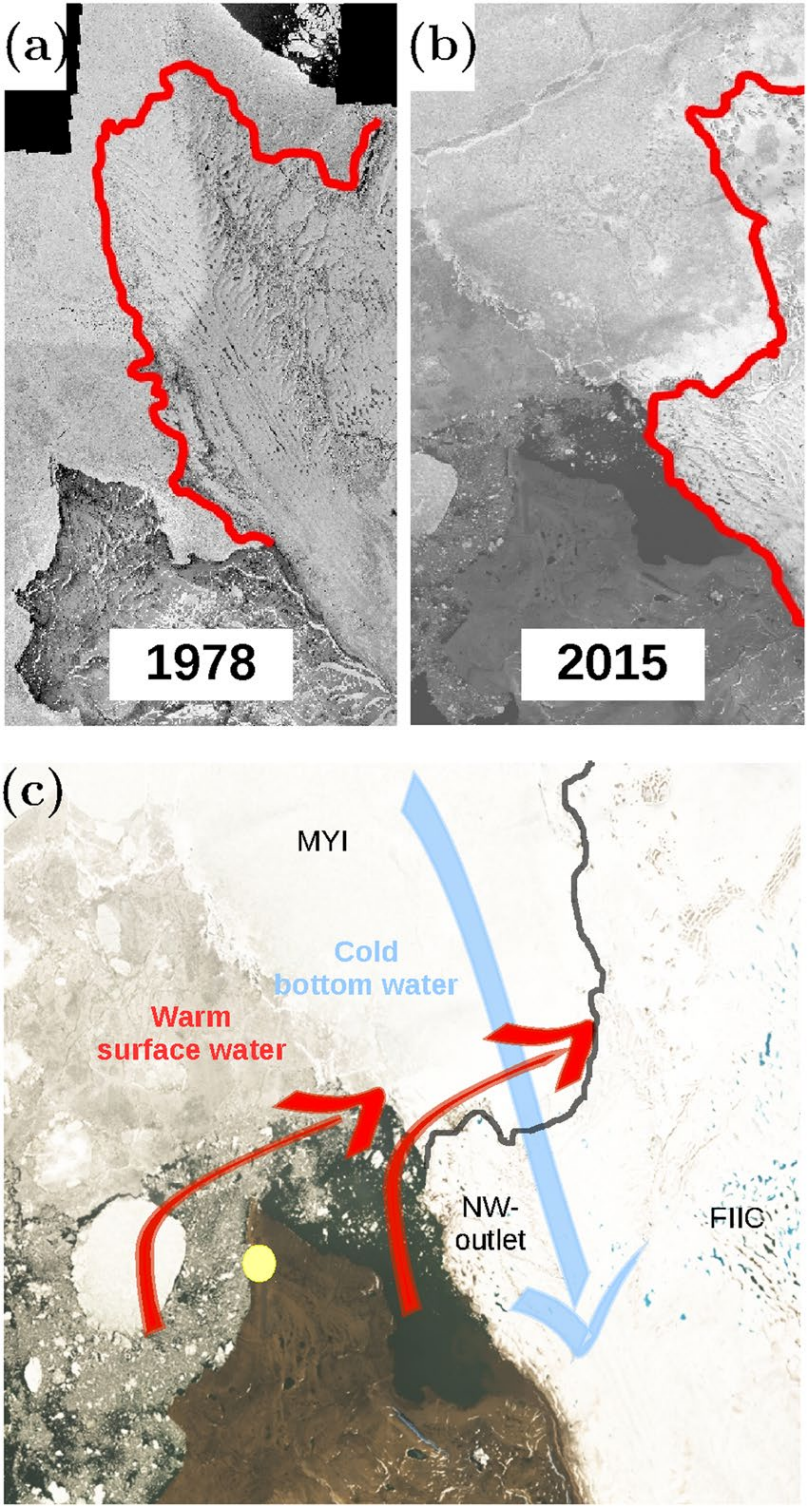
Melt due to open water. (**a**) FIIC in 1978 and **(b)** 2015. **(c)** Conceptual figure showing transport of warm surface water (red) and relatively cold bottom water (blue) below the ice shelf. **(a)** is based on orthophotograph from Korsgaard *et al*.^44^ (http://www.nature.com/articles/sdata201632) licenced under CC BY 4.0 (https://creativecommons.org/licenses/by/4.0/) (**b**) and (**c**) are enlargements of the Landsat 8 satellite image shown in Fig. 1a (Landsat 8 data compiled by the U.S. Geological Survey and processed as in Fig. 1). The location of Villum Research Station (yellow bullet) and the NW-outlet glacier are shown. Relatively thin permanent ice cover and thick multi-year ice (MYI) cover the western and eastern portion of the area, respectively.

A comparison of orthorectified aerial photographs from 1978 with present day conditions show a significant decrease of a long and narrow ice tongue by more than 10 km with considerable variability of the ice extent over time (Figs 6 and S2). The current position of the terminus represents a minimum extent of the ice tongue, comparable with the extension in 2004, since 1961. Joughin *et al*.^32^ note that the NW-outlet glacier has surge-type behavior, with large ice velocities in 2000–2001 reaching 300–350 m yr^−1^ followed by a decrease to about 60 m yr^−1^ in 2005 where the glacier returned to its more quiescent phase. This behavior is supported by radar- and laser satellite altimetry data supplemented with surface mass balance modelling, which combined reveal dynamically driven thickening of the northwestern part of FICC^33^.

### A floating ice shelf at the NW-outlet glacier

Our echo soundings along transects I and III showed that the depth increased abruptly from ~35 to 50–70 m, when sailing away from the glacier, and this could be indicate either a grounded vertical face or a a floating glacier. The presence of high saline bottom water at the deepest part along the terminus (i.e. transect III near land) could not be explained by mixing with nearby relatively low-saline water masses along or in front of the terminus. No sufficiently deep connections were observed west off the terminus to explain a deep transport from station NS to the bottom water at transect III (Fig. 1d). This suggests that either (i) high saline bottom water was spilled over the shallow areas during windy periods or, as we argue below, that (ii) bottom water from further out on the shelf intrudes below the ice shelf to the deep area at transect III. The observed *in situ* freezing temperatures between 30 and 50 m in front of the glacier at transect III could be explained by modification of bottom water from station NS by heat loss due to melting of glacial ice below the ice shelf. Short-term variability of bottom water observed at transect III between 13 and 21 August, when temperature increased by ~0.1 °C (Fig. 4c), could also be explained by a renewal of warmer bottom water below the ice shelf. Thus, this line of observations together with the surface elevation of the glacier discussed above, all indicate a floating ice shelf at the NW-outlet glacier.

### Melting and circulation below the ice shelf

The presence of relatively warm bottom water (>−1.4 °C compared to freezing temperatures below −1.7 °C) near FIIC showed that coastal water masses may cause significant bottom melt below the ice shelf. A comparison between bottom water masses and observations from station YK175 located on the continental slope off FIIC (white bullet in Fig. 1a) show that the temperature at YK175 below 70 m was significantly lower than observed at station NS (Fig. 4a).

Based on these observations we suggest that intrusions of coastal water masses below a ~45 m thick ice shelf result in ice melting and cooling of water below the ice shelf to the freezing point of seawater. Subsurface freezing temperatures indicate freezing, and possibly refreeze of melted ice, below the glacier. This cold-water phenomenon, observed at glaciers around Antarctica^34^, has not been observed elsewhere at tidewater outlet glaciers around Greenland. Shelf water masses only appear in the deepest parts, suggesting that intrusion takes place in relatively deep channels (i.e. >70 m) on the seafloor further towards the east.

### Freshwater content in the outflow region

Surface layer temperature and salinity were analyzed in the upper 20 m of the water column from locations off VRS, at station NS near the MYI and at transect III at the inner part of the NW-outlet glacier (Table 1). The area was, in general, characterized by a shallow 2–6 m deep surface mixed layer, with surface temperatures and salinities in August ranging between 0.0 and 2.3 °C and 6 and 12, respectively. A relatively large freshwater content (FWC, Methods) of ~3.6 m in the upper 10 m characterized the area and may be representative of the outflowing region between FIIC and HTL. A somewhat larger FWC of 4.2 m at transect III reflected the influence from glacier melting and runoff from the meltwater river outlet at the coast. CTD-profiles obtained during April and May (before surface melt commenced) off VRS were characterized by water temperatures of ~−1.0 °C and a significantly lower freshwater content of 1.6–1.9 m. Thus, the seasonal change in FWC was about 2 m. A similar large seasonal change in FWC was found in the East Greenland Current (EGC) just south of Fram Strait^35^, and this shows that a significant amount of meltwater is mixed into the surface layer in this region.

**Table 1:** Surface layer characteristics.

St. ID	Date [YYYYMMDD]	longitude	latitude	Depth [m]	SST [°C]	T_0–10m_ [°C]	SSS	S_0–10m_	FWC [m]	Location
90	20150810	−16.851	81.623	23	0.99	0.25	6.9	21.6	2.9	off VRS
154	20150818	−17.023	81.643	62	2.32	1.21	12.21	19.6	3.6	off VRS
166	20150821	−16.260	81.604	77	2.00	0.71	9.42	17.8	4.2	Tr. III
199	20150821	−17.124	81.723	88	0.06	−0.25	5.57	19.6	3.6	Station NS
15	20150419	−16.805	81.624	>20	−1.08	−1.30	20.10	25.6	1.6	Spring, off VRS

CTD observations at stations located west off Villum Research Station (VRS), the northern station near the multiyear ice (station NS), Transect III and a profile from spring 2016, respectively. Depth of the station, sea surface temperature (SST) and salinity (SSS), average temperature, salinity and freshwater content (FWC) in the upper 10 m are shown.

### Melt rates in the open water period

In order to evaluate glacier melt in the surface layer we assess the energy budget. Our measurements showed that surface water temperature in the upper 10 m increased by more than 2.5 °C in the open water areas near the glacier compared to early spring conditions (Table 1). This corresponded to a change in the seasonal heat content (Methods) of 103 MJ m^−2^. The average incoming solar insulation from 1–25 August was 164 W m^−2^ corresponding to a total solar heat flux of 333 MJ m^−2^ during the open water period (assuming an albedo of 6%). Measurements of light attenuation off VRS showed that the euphotic zone (i.e. 1% light level) was only ~6 m deep, in accordance with the observed shallow summer thermocline. Thus, solar heating during the short open water period in August in front of FIIC can explain the observed change in heat content between early spring and late summer. The incoming solar heat flux on top of the glacier during this period of ~106 MJ m^−2^ (assuming an albedo of 70%) is comparable to the increased energy content in the surface layer, and corresponds to the energy required for melting 0.4 m m^−2^ of ice.

The direct melt from a surface layer with an average temperature of 0.7 °C (Tr. III, Table 1), estimated according to Neshyba and Josberger^36^ (Methods), was 0.5 m month^−1^. Including the influence from wave erosion, calculated from wind speed and direction^37, 38^, increased this value by an order of magnitude to 0.7 m day^−1^. The surface temperature increased from near freezing temperature during the open water period and, therefore, these melt rates should be reduced by about 50% (e.g. assuming a linear temperature increase of the surface layer). Thus, melt from the surface layer resulted in ~10 m month^−1^ during the short open water period in August.

### Seasonal sub-surface melt rates

The temperature at the CTD-mooring was relatively constant in May and June (~−1.5 °C) while the gradual increase in July could be related to open water areas north of the MYI cover as evident from MODIS satellite imagery from 22 June (Fig. 5). The corresponding melt rates (Methods, Eq. 3) increased by a factor of ~6 from 0.03 in May to 0.17 m month^−1^ in August 2015 with an average melt rate during the whole period of 1.2 m yr^−1^.

The temperature from the CTD-mooring 21 August 2015 (−1.0 °C) was compared with temperatures at station NS (−1.2 °C) and at transect III (−1.5 °C) at the corresponding density level (σ_t_ = 24.15 kg m^−3^) and this showed that the temperature near the glacier, at ~13 m depth, was significantly colder than at the mooring. This could be explained by heat loss for melting of glacier ice. The melt rates calculated from the CTD-mooring, therefore, represent an upper bound of melting below the upper halocline. Thus, subsurface melting at this depth level is relatively small when compared to melt from the surface layer in the open water period. Surface heating in August increased surface water temperature to above 2 °C in front of the glacier corresponding to a surface melt rate in the upper 5 m of ~0.8 m month^−1^, whereas melt rates calculated at station NS corresponded to relatively low values of 0.1 m month^−1^ in the upper few meters (due to the MYI cover) and ~0.4 m month^−1^ at ~5 m depth (Fig. 5d). Melt rates calculated from the CTD-profiles near the terminus from May 2015 showed very small values (<0.02 m month^−1^) in the upper 10 m (Fig. 5d). This implies that melting of glacier ice in the upper 10 m mainly occurred during the short period with ice breakup and, in particular, high surface water temperatures in the open water area at the terminus made a comparatively large contribution to the annual melt.

### Ice foot morphology

Melting of the glacier front by the relatively warm surface layer resulted in the formation of a ~40 m deep and ~40 m wide ice foot along sections of the glacier front (Figs 5e and S3). The width of the ice foot at transect III was 38 m and it was ~35 m deep. At a distance of 28 m from the terminus the ice foot was 15 m deep. This corresponded to a lateral melt (δx, Fig. 5e) of ~10 m (i.e. 38–28 m) of the ice foot, equivalent to about 8 years of melt with the sub-surface melt rate of 1.2 m yr^−1^. The relatively large melt of the ice foot in the upper 5 m could be explained by the warm surface layer in August^36, 39^. At transect I, the ice foot was only ~15 m wide, indicating that here calving had eroded the ice foot and, hence, we speculate that calving may also occur due to the “footloose” mechanism^40^ operating on large ice bergs where buoyancy forces from a large ice foot lead to upward calving. We witnessed an upward calving event close to the terminus. In addition, several icebergs in the area had a ‘tilted’ orientation (Fig. S3).

### Open water and energy balance at FIIC

The climatological annual averaged sea ice extent in the Arctic Ocean has decreased by 14% between the period 1963 and 1998 and by 27% since 1950 considering the minimum sea ice extent in 2012^41^. Observations at Station Nord (located at VRS) between 1950 and 1964 show that the area was covered by sea ice throughout the year^42^. Higgins^30^ noted that extensive open water leads were visible close to the terminus on aerial photographs (from plane) in 1978. The presence of open water has been visually observed around Station Nord every summer in recent years.

Our results show that the duration of open water increases heat content in the surface layer significantly and transport of warm surface water may, therefore, impact the NW-outlet glacier. Melting of the disintegrated ice tongue in front of the NW-outlet during the last decades and the subsequent replacement of the ice tongue with MYI can, therefore, be explained by transport of warm surface water from open water areas (Fig. 6c). The role of surface melt on the glacier would increase non-linearly when the glacier tongue starts to break up because of increased melt in cracks and channels around the calved icebergs. Such large channels and cracks into the ice tongue were observed along the terminus during August 2015.

Relating the melt rates, inferred from the 2015 observations, with ice dynamics reveal that annual ice flow can easily be balanced by short periods of open waters. Estimating the heat required for melting the annual ice flux of the glacier tongue from the NW-outlet based on the ice flow (60 m yr^−1^), width (~10 km) and depth (~40 m) of the glacier tongue yield ~7.4 PJ yr^−1^. The ocean heat transport in the surface layer (containing 103 MJ m^−2^) towards the terminus with a length of ~10 km and assuming an ocean current of 1 cm s^−1^ corresponds to an energy flux of 0.9 PJ day^−1^. These values are rough estimates based on our current knowledge of the ice flow and ocean currents estimated from the relatively low salinity and observed tidal motion of ice floes. A transport towards the glacier would cause melt along the terminus and in the wide cracks into the glacier. Some of the heat will also be used for melting sea ice and convergence in front of the glacier would redirect currents along the terminus and, therefore, only a portion of the heat flux calculated above would be available for melting glacier ice. However, transport of warm surface water towards the glacier during a few weeks would equal the energy needed for melting the annual ice flow from the NW-outlet and is comparable to the energy flux from solar insulation in August (i.e. ~106 MJ m^−2^) above a corresponding area (i.e. 100 km^2^) of ~10 PJ. Thus, heating of the ice free surface layer is a main component of the energy budget around the NW-outlet.

## Conclusion

Hydrographic measurements at the NW-outlet, a major tidewater outlet glacier from Flade Isblink ice cap in northeastern Greenland, showed that the outflowing region was characterized by warm surface water above 1 °C and low salinities, corresponding to a freshwater content of ~3.6 m (using a reference salinity of 30.61). Water mass properties at the bottom of the terminus and ice surface elevation data supported that the NW-outlet has a floating ice shelf. Bottom water at the terminus was super-cooled or very near the freezing point, providing a strong indication that net freezing took place at the glacier ice-ocean interface. During the short (~1 month) open water period the increased heat content of the surface layer was estimated to account for up to ~10 m of ice melt per month. The presence of open water in front of the terminus and the associated solar heating of the surface layer were found to be a major contribution to the annual melt. Reduction in sea ice cover in front of tidewater outlet glaciers along the north coast of Greenland could, therefore, result in a significant increase of ocean heat transport towards tidewater outlet glaciers.

## Methods

### Hydrographic and meteorological measurements

The field campaign was carried out from two inflatable boats (Zodiac) equipped with a winch and a SeaBird Electronics SBE19plus SEACAT profiler measuring conductivity, temperature and depth (CTD) and a PAR Licor sensor. The CTD-mooring off VRS (81°36.839′N, 16°45.888′W) was a SeaBird-Electronics SBE37SMP Microcat in a frame deployed on the bottom (sensors ~0.2 m above bottom) at 13.5 m depth (Station M, Fig. 1b). The instrument was only calibrated before deployment. However, the observed temperature and salinity changes are at least two orders of magnitude larger than the instrument drift (specified by the manufacturer to 0.003 mS/m and 0.0002 °C per month). The applied CTD profile from 19 April 2015 was taken at 81°43.415′N, 17°31.861′W. The CTD’s were calibrated by the manufacturer before the field campaign. The CTD-profile from the Norwegian FRAM 2014/15 sea ice drift expedition was obtained from the research hovercraft SABVABAA 5 on the continental slope off FIIC (station YK175, 5 July 2015, 82°11.6′N, 10°13.8′W, Fig. 1a). Salinity measurements are reported as practical salinity.

Meteorological data was measured at the VRS weather station and incoming short wave radiation, wind speed and wind direction were measured every 30 minutes.

### Bathymetry and satellite data

Garmin echo sounders (Garmin echoMAP 50dv) were mounted at the rear of the two zodiacs and about 10 cm below water level to continuously record bottom depth. Map of northeastern Greenland (Fig. 1) was based on IBCAO^43^. A Landsat 8 satellite image from 12 August was used for digitizing high-resolution coastline and, glacier- and MYI edges. A 1978 orthophoto and digital elevation model^44^ were used to digitize the western ice tongue at FIIC and to extract a glacier surface flow line profile with a horizontal resolution of 25 m which subsequently was low-pass filtered using a 675 m window.

### Heat and freshwater content

The seasonal change in heat content (HC_s_) was calculated as:

1 $$H{C}_{s}=\int \rho {c}_{p}\Delta Tdz$$

where ρ and c_p_ are the density and heat capacity for seawater, respectively. The temperature difference between *in situ* summer temperature (T) and early spring temperature, determined as ΔT = (T − T_spring_), correspond to the change in heat content during the summer season. The heat content is integrated in the upper 10 m and *z* the vertical coordinate.

Freshwater content (FWC) was calculated by integrating the difference of *in situ* salinity and a reference salinity of 30.61, corresponding to the measured salinity at 20 m at station NS:

2 $$FWC=\int \frac{{S}_{ref}-S}{{S}_{ref}}dz$$

### Meltwater rate

The meltwater rate (V_m_, m yr^−1^) at the ice-ocean interface was calculated according to Neshyba and Josberger^36^ as follows:

3 $${{\rm{V}}}_{{\rm{m}}}=2.78\,\Delta {\rm{T}}+0.47\,\Delta {{\rm{T}}}^{2}$$

where ΔT is the difference between seawater and the freezing point temperature.

Melt from wave erosion (V_w_, m s^−1^) was calculated from the formula provided by White *et al*.^37^:

4 $${V}_{w}=a{(\frac{{z}_{r}}{{H}_{w}})}^{b}(\frac{{H}_{w}}{{T}_{w}})\Delta {T}^{2}$$

where *a* and *b* are constants (with values of 1.46 · 10^−4^ and 0.2, respectively), *z*_*r*_ is the roughness height of the ice surface (~1 cm) and H_w_ and T_w_ are significant wave height and period, respectively. Significant wave height and period were calculated from half hourly measurements of wind speed assuming a fetch of 2 km, corresponding to the estimated open water area in front of the glacier. Only wind forcing from directions between 150 and 330 degrees, corresponding to wind directions towards the glacier, were considered. Significant wave height and period were calculated according to Kahma and Calkoen^38^.

## Electronic supplementary material

Supplementary Information
