# Supplementary material for: Sea ice breakup and marine melt of a retreating tidewater outlet glacier in northeast Greenland (81°N)
Source: Sci Rep. 2017 Jul 10;7:4941. doi: 10.1038/s41598-017-05089-3 (PMC5503942; doi:10.1038/s41598-017-05089-3)
Supplement: Supplementary file 1 — Supplementary Information [file 41598_2017_5089_MOESM1_ESM.pdf]

Supplementary Information

Sea ice breakup and marine melt of a retreating tidewater outlet glacier in northeast Greenland (81° N)

Jørgen Bendtsen[1,2,*], John Mortensen[3], Kunuk Lennert[3], Jens Ehn[4], Wieter Boone[4], Virginie Galindo[4], Yubin Hu[4], Igor A. Dmitrenko[4], Sergey A. Kirillov[4], Kristian K. Kjeldsen[5,6], Yngve Kristoffersen[7], David Barber[4] & Søren Rysgaard[1,3,4]

[1] Arctic Research Centre, Aarhus University, 8000 Aarhus, Denmark.

[2] ClimateLab, Symbion Science Park, Fruebjergvej 3, 2100 Copenhagen O, Denmark.

[3] Greenland Climate Research Centre, Greenland Institute of Natural Resources, PO Box 570, 3900 Nuuk, Greenland.

[4] Center for Earth Observation Science, CHR Faculty of Environment, Earth, and Resources, University of Manitoba, 499 Wallace Building, Winnipeg, MB R3T 2N2, Canada.

[5] Centre for GeoGenetics, Natural History Museum, University of Copenhagen, Øster Voldgade 5-7, 1350 Copenhagen K, Denmark.

[6] Department of Earth Sciences, University of Ottawa, Ottawa, Ontario K1N 6N5, Canada

[7] Department of Earth Science, University of Bergen, Bergen, Norway

[*] Corresponding author at: ClimateLab, Symbion Science Park, Fruebjergvej 3, DK-2100 Copenhagen, Denmark, Tel: +45 2167 1535.

E-mail address: jb@climatelab.dk (J. Bendtsen).

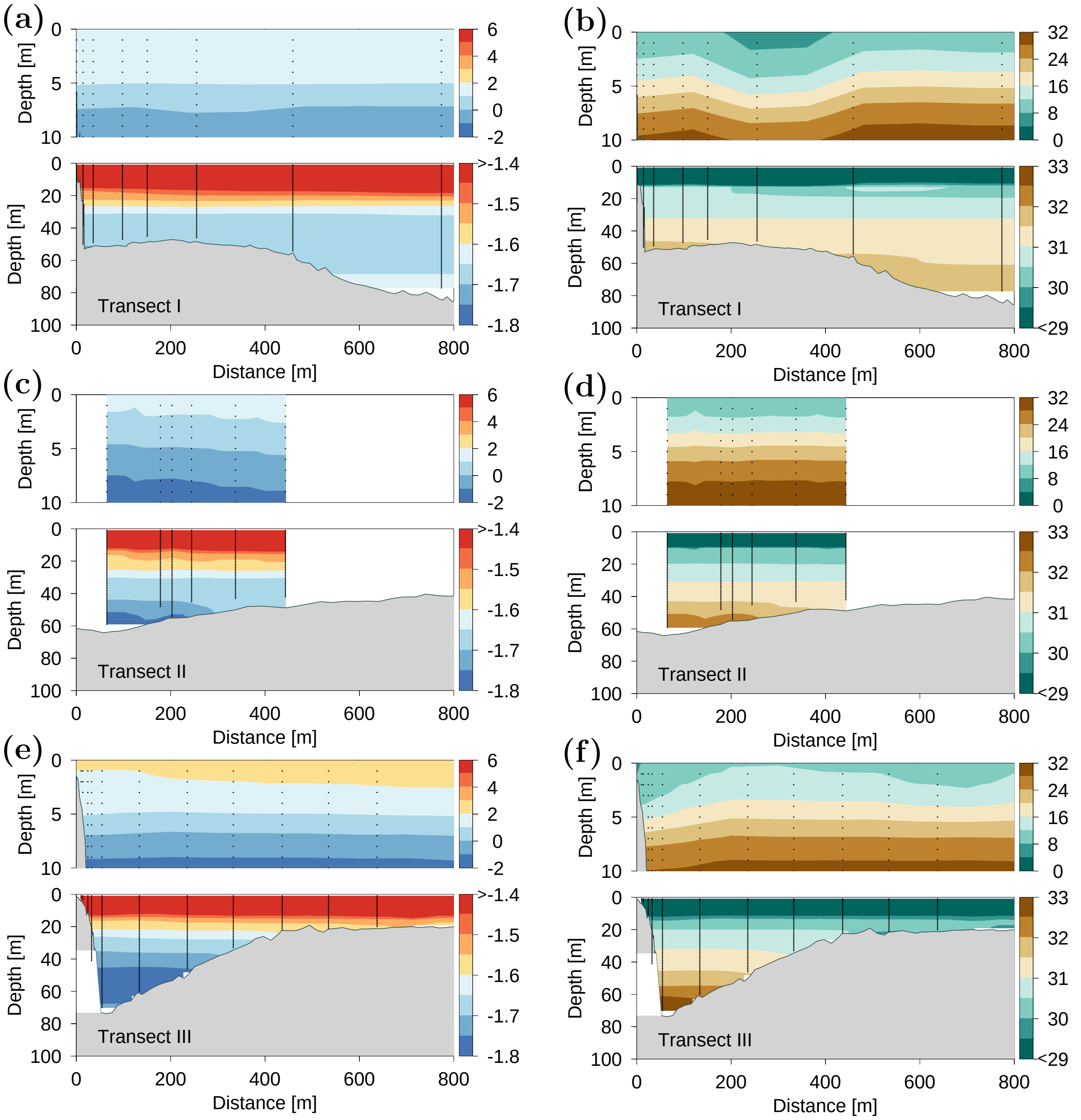

Figure S1 Transects of temperature **(a)**, **(c)**, **(e)** and salinity **(b)**, **(d)**,**(e)** versus distance from the glacier. The upper panel show the upper 10 m and the lower panel show the whole water column. Note the differenct color scales. All data was obtained 21 August 2015 and stations are shown with dotted lines. The glacier is gray shaded in the upper part of transect I and III.

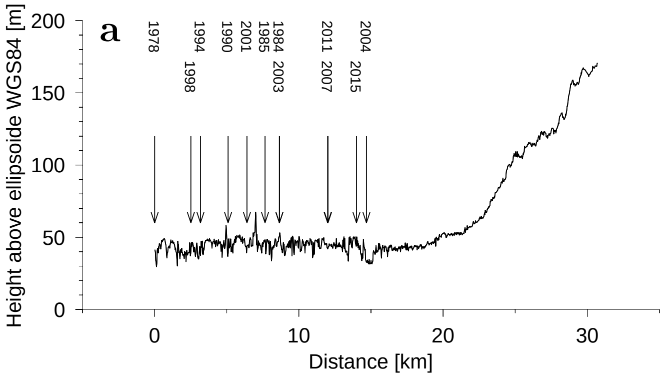

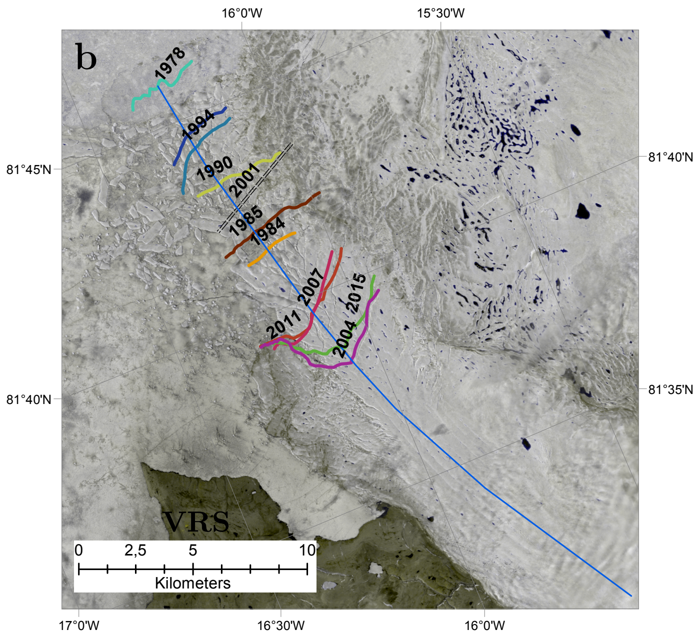

Figure S2 a) Glacier surface flowline profile (blue line in panel b) and locations of the northermost extents of the ice tongue since 1978. Distance is from the extension in 1978. b) ASTER satellite image from August 11th 2003 retrieved from U.S. Geological Survey Earth Resources Observation and Science (EROS) Center showing the disintegration of the ice tongue. The northernmost locations of the terminus, identified from the 1978 orthophoto and satellite images, are marked with colors and years. Note that the extent in 2001 is a minimum estimate due to the extent of the available Landsat 7 satellite imagery. The location of VRS is indicated

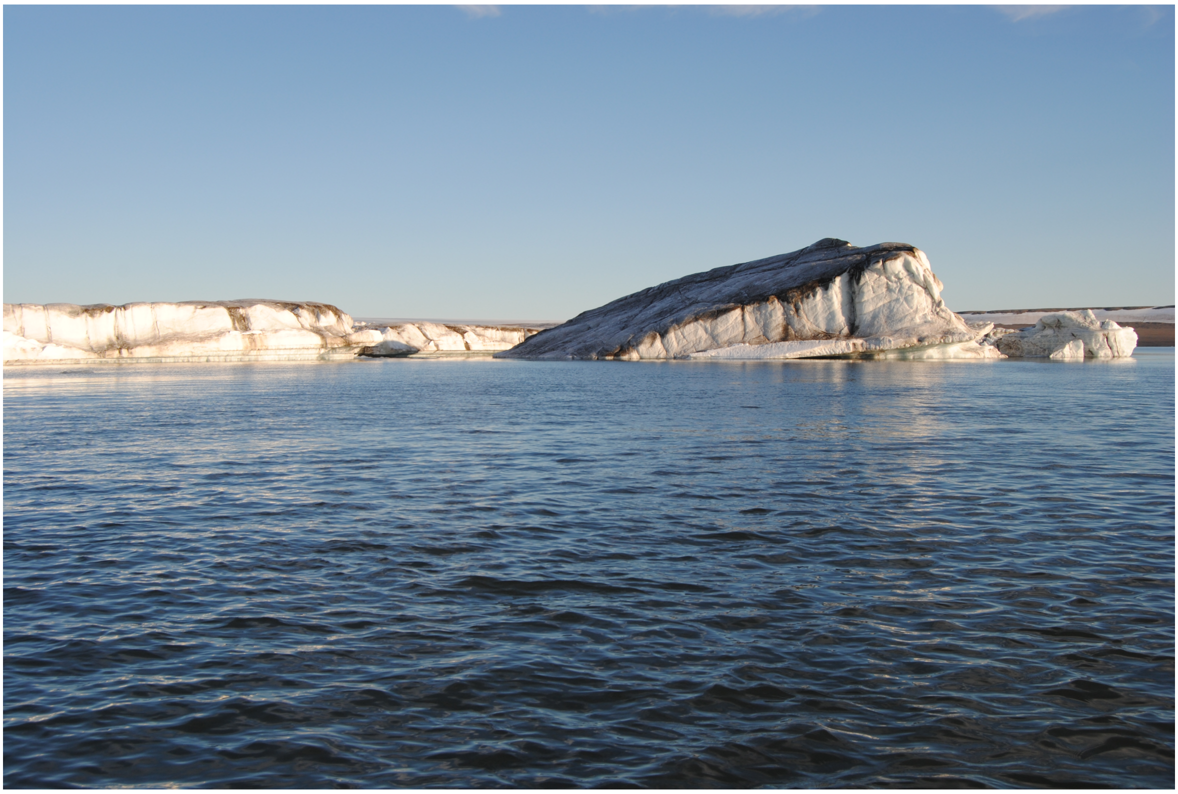

Figure S3 Tilted iceberg, possibly grounded, near land at the terminus of Flade Isblink. The remains of the ice foot is visible at the right side of the ice berg. The height of the terminus seen in the background is about 4-6 m above sea level. Photo by J. Bendtsen, August 2015.
